# Three-Dimensional Deformation Calculation of Wind Tunnel Flexible Wall Using Orthogonal Beam Function

**DOI:** 10.3390/ma18153593

**Published:** 2025-07-31

**Authors:** Xiuxuan Yang, Yueyin Ma, Guishan Wang, Can Yang, Chengguo Yu

**Affiliations:** Facility Design and Instrumentation Institute, China Aerodynamics Research and Development Center, Mianyang 621000, China; xiuxuanyang@outlook.com (X.Y.); mayueyin@cardc.cn (Y.M.); wangguishan10@nudt.edu.cn (G.W.); yangcan@cardc.cn (C.Y.)

**Keywords:** transonic/supersonic wind tunnel, flexible wall, large-deflection deformation, elliptic integral solution, orthogonal beam function

## Abstract

Transonic/supersonic wind tunnels are indispensable equipment for advanced aircraft to operate across subsonic, transonic, and supersonic regimes. The deformation of the flexible nozzle is the key to accurately controlling the Mach number of transonic wind tunnels. However, solving the deformation of flexible wall plates remains challenging due to the highly nonlinear relationship between wall loading and deformation, as well as the lack of simple yet effective mathematical models under complex boundary conditions. To accurately describe the deformation of flexible wall plates and improve computational efficiency, this study systematically investigates the deformation characteristics of flexible walls in two orthogonal directions and proposes an orthogonal beam function (OBF) model for characterizing small-deflection deformations. For large-deflection deformations in a flexible wall, an elliptic integral (EI) solution is introduced, and the OBF model is correspondingly modified. Experimental validation confirms that the OBF model effectively describes large-deflection deformations in a flexible wall. This research contributes to solving large-deflection deformation in flexible wall plates, enhancing both computational efficiency and accuracy.

## 1. Introduction

Flexible nozzles are a key technology of Mach number control in transonic/supersonic wind tunnel design. By controlling the deformation of the flexible wall plate within the flexible nozzle, variable Mach number conditions can be achieved, significantly enhancing wind tunnel efficiency and experimental performance [1,2,3]. Numerous scholars have conducted detailed investigations into the deformation [4,5,6] and buckling [7,8] of rectangular thin plates under various boundary conditions, providing a solid theoretical foundation for analyzing the large deflection deformation of flexible-wall nozzles. However, the deformation of flexible wall plates constitutes a large-deflection problem, characterized by a highly nonlinear relationship between applied loads and deformation. Furthermore, the lack of simple yet accurate mathematical models under complex boundary conditions makes solving this problem particularly challenging. Existing modeling theories and methods, such as beam theory and finite element analysis, suffer from limitations in computational efficiency or solution accuracy. To accurately describe the deformation of flexible walls and improve the solving efficiency, it is necessary to explore an efficient, simple, and high-precision modeling and solving method based on the mechanism of large deflection deformation of flexible walls.

Structural deformation problems are generally categorized into small-deflection and large-deflection deformations [9,10]. The deformation of flexible wall plates falls under the category of large-deflection deformation of thin plates. Currently, the most commonly employed analytical and computational methods in engineering practice are numerical and analytical solution approaches. Numerical solutions are typically employed during the post-design phase, where commercial software such as finite element analysis or multibody dynamics simulation tools is used to analyze and validate predefined nozzle models. Chen et al. [11] investigated the computational methodology and dynamic characteristics of a single-support semi-flexible nozzle using rigid-flexible coupled dynamics software. They proposed an axial free-extension technique for flexible wall panels and conducted wind tunnel tests to achieve continuous Mach number variation, validating their design approach through flow-field measurements. Similarly, Nie et al. [12,13] explored the dynamic simulation of semi-flexible nozzle mechanisms by integrating rigid-flexible coupling theory with numerical simulation tools, including Mechanical System Dynamics Automatic Analysis (ADAMS) and NASA Structural Analysis (NASTRAN). While FEA-based numerical methods can accurately capture the mechanical behavior of flexible nozzles and yield high-precision results, they suffer from several limitations: complex modeling procedures, long computational cycles, high resource demands, and low analysis efficiency. These drawbacks hinder rapid iterative design between structural and aerodynamic optimization, making them unsuitable for real-time nozzle profile monitoring during wind tunnel commissioning and operation.

Analytical solutions, primarily based on small-deflection linear beam theory, provide approximate calculations for structural deformation problems. Milam W. E. et al. [14] analyzed the deformation of a flexible wall using a piecewise linearization approach according to small-deflection beam theory. Wu et al. [15,16] employed cubic spline curves to describe nozzle profiles, taking height coordinates of interpolation points as design variables for optimizing the contour of a 0.6 m continuous transonic wind tunnel nozzle. They developed a restart optimization algorithm based on Gaussian process modeling, which improved the global characteristics of gradient algorithms while reducing the number of aerodynamic evaluations, thereby enhancing computational efficiency and flow field quality. For the large deflection deformation of beams, methods such as the Adomian decomposition method [17], the pseudo-rigid-body model [18], and the elliptic integral method [19] are commonly employed for deformation calculations. Among these, the elliptic integral method exhibits high accuracy and efficiency, demonstrating significant advantages in solving large deflection beam problems. Kimball et al. [20] derived an elliptic integral solution for a beam with a single inflection point under combined force and moment loading. Holst et al. [21] further applied the elliptic integral method to solve the large deflection deformation of fixed-guided beams. Current research indicates that while the linear beam approximation method offers computational simplicity for large-deflection flexible wall panel problems, it neglects deformation along the transverse direction, inevitably introducing certain approximation errors [22,23].

The deformation of flexible wall plates actually constitutes a large-deflection problem of rectangular thin plates. To elucidate rectangular plate deformation under various boundary conditions, numerous scholars have conducted extensive research based on plate and shell deformation theories [5,6,24,25]. Current theoretical foundations for large-deflection deformation problems include the Donnell theory for cylindrical shell structures [26], the Von Karman large-deflection bending theory for thin plates [27], and mechanics-based analytical methods derived from Hamilton’s principle [24], among others. These theories provide a solid theoretical basis for studying the large-deflection deformation of flexible-walled nozzles. However, due to the complexity of plate and shell deformation theory, it is difficult to apply it directly to the engineering design of a flexible wall. At present, there are no direct and simple methods to solve the deformation problem of a rectangular thin plate under the specific complex boundary conditions of a flexible nozzle. Therefore, more in-depth research is required in theoretical modeling and large-deflection solutions to meet the design and analysis requirements of flexible nozzles.

This study investigates theoretical computational methods for large-deflection deformation in wind tunnel flexible nozzles. First, an OBF for rectangular thin plates was established based on small-deflection beam theory, followed by differential analysis of the OBF model. Subsequently, the elliptic integral method was employed to solve the large-deflection deformation problem along the principal bending direction of rectangular thin plates. Building upon these foundations, a three-dimensional computational model for large-deflection deformation of rectangular thin plates was developed using the OBF model. The proposed model was validated through both experimental testing and numerical simulations. Results demonstrate that the OBF model can effectively address large-deflection deformation problems in flexible wall panels, enabling accurate three-dimensional deformation calculations.

## 2. OBF Model Under Small Deflection Deformation

### 2.1. Flexible Wall Structure and Stress Analysis

**Figure 1:**
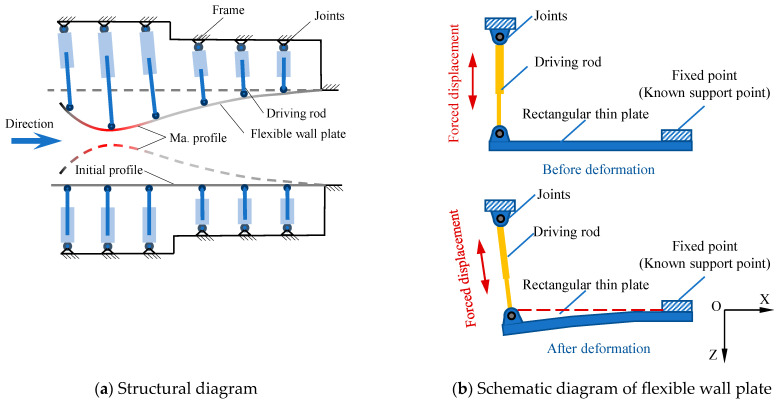
Structural diagram of multi-fulcrum flexible wall nozzle.

The structure of a typical flexible wall nozzle is shown in Figure 1a, which mainly includes a flexible wall plate, driving rod, frame, and other components. In the initial installation state, the flexible wall plate is flat, and when working, elastic deformation occurs in the flexible wall plate under the forced displacement of the driving rod, forming the designed Mach number nozzle profile. A flexible wall nozzle with multiple fulcrums is constrained at one end, free at two opposite sides, and constrained by forced displacement under the action of multiple fulcrums in the middle. The deformation problem of a flexible wall nozzle is a large deflection deformation problem of rectangular thin plates with complex boundary conditions. Considering the constraint conditions remain consistent between every two adjacent support points, the flexible wall plate is partitioned into several single rectangular thin plates by using the supporting points as boundaries (Figure 1b), and the elastic deformation of rectangular thin plates with known boundaries is emphatically studied.

### 2.2. Establishment of Orthogonal Beam Function of Flexible Wall Plate

**Figure 2:**
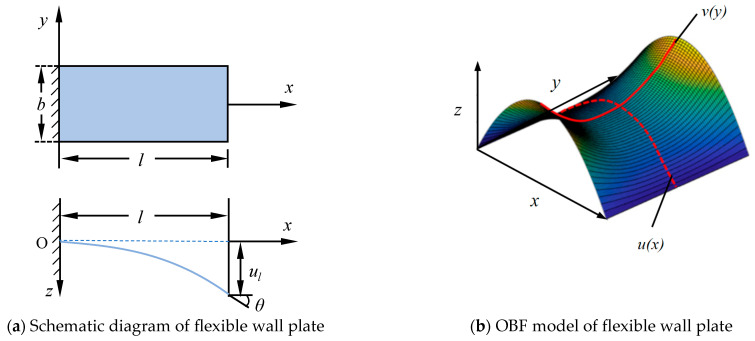
Schematic diagram of boundary conditions of flexible wall plate and OBF model.

As shown in Figure 2a, driven by the driving rod, the flexible wall plate bends along the axis of the wind tunnel, and the deformation along the transverse direction results from the shear strain induced by the flexible wall plate’s bends. The length of the flexible wall plate is *l*, the transverse is *b*, the forced rotation angle of the free end is θ, and the forced displacement is *u_l_*. Due to the forced constraint of the driving rod and its hinge at the support point, the flexible wall plate exhibits negligible deformation along the transverse direction. Consequently, the maximum deformation along the transverse direction is generally located in the middle of two adjacent support points. To model this behavior, we proposed an orthogonal beam-function model that employs two orthogonal functions to represent deformations along the primary bending and transverse directions, with the overall deformation obtained through superposition. As illustrated in Figure 2b, $u\left(x\right)$ represents the beam function in the primary bending direction, while $v\left(y\right)$ corresponds to the beam function in the transverse direction. The progressive color gradient from blue to red indicates increasingly minor to major deformation magnitudes, respectively. The superposition of these two functions demonstrates that the transverse deformation profile remains identical at both the fixed and constrained-displacement boundaries, while the central region develops characteristic saddle-shaped geometry.

To construct the OBF model, a linear beam with small deflection is selected as the beam function in the primary bending direction, and the OBF model is proposed and verified. Then, the accurate solution of large deflection deformation in the primary bending direction is studied. For the primary bending direction, the system can be modeled as a beam with one end fixed and the other end subjected to prescribed transverse displacement and rotational constraints. To simplify the analysis, only unidirectional constraints are considered, with the assumption of zero bending moment at the free end. As shown in Figure 2a, the beam coordinates take the fixed end as the origin O, the primary bending direction is the *X* direction, and the forced displacement direction is the *Z* direction.

For elastic beams with small deflection deformation, the boundary conditions are

(1) $${\left(u\right)}_{x=0}=0$$

(2) $${\left(\frac{\partial u}{\partial x}\right)}_{x=0}=0$$

(3) $$u_{x=l}=u_{l}$$

(4) $${\left(\frac{\partial u^{2}}{\partial^{2}x}\right)}_{x=l}=0$$

where u is the deflection deformation of the elastic beam, *u_l_* is the forced displacement of the free end (Figure 2a), Equations (1) and (2) represent the deflection and rotation angle of the fixed end as 0, and Equations (3) and (4) represent the deflection and bending moment of the free end as 0. For elastic beams with small deflection, the simplified curvature is

(5) $$\frac{1}{\rho \left(x\right)}=\frac{\partial u^{2}}{\partial^{2}x}=-\frac{M\left(x\right)}{EI}$$

where ρ is the curvature radius, $M\left(x\right)$ is the moment of the beam at *x*, *E* denotes the elastic modulus, and *I* represents the moment of inertia of the cross-section. The deformation along the *X* direction after the beam deformation is not considered, and the bending moment is

(6) $$M\left(x\right)=F\left(l-x\right)$$

where *F* is the force on the free end, and *l* is the length of the beam.

According to the boundary condition Equations (1)–(4), the deflection equation is solved as follows:

(7) $$u\left(x\right)=-\frac{\delta }{2l^{3}}x^{3}+\frac{3\delta }{2l^{2}}x^{2}$$

where δ is the deflection on the free end.

As the length-thickness ratio of the flexible wall plate is more than 20 and shear force is ignored, according to the geometric equation of the Euler beam, the relationship between principal strain and curvature of the elastic beam is the following:

(8) $$\varepsilon \left(x\right)=-\frac{h}{2\rho \left(x\right)}$$

where *h* is the thickness of the beam. From Equations (5) and (8), the $\varepsilon \left(x\right)$ is given as follows:

(9) $$\varepsilon \left(x\right)=-\frac{h}{2}\cdot \frac{\partial u^{2}}{\partial^{2}x}$$

According to the relationship between tension and compression strain and shear strain on the upper and lower surfaces of a rectangular thin plate, the shear strain of the flexible wall plate $\gamma \left(y\right)$ is

(10) $$\gamma \left(y\right)=\mu \varepsilon \left(x\right)$$

where μ is the Poisson’s ratio of the flexible wall plate. Since the primary bending direction is described using beam functions and the variation of principal strain $\varepsilon \left(x\right)$ along the transverse direction is neglected, it can be assumed that the shear strain $\gamma \left(y\right)$ also remains constant across the transverse direction at the same cross-section.

Considering the deformation in the transverse direction, the flexible wall plate has a left-right symmetrical structure. Assuming that the displacement of the middle section is zero and the strain of each point along the transverse direction is equal, the boundary conditions of the deformation in the transverse direction are the following:

(11) $${\left(v\right)}_{y=0}=0$$

(12) $${\left(\frac{\partial v}{\partial y}\right)}_{y=0}=0$$

(13) $$\frac{\partial v^{2}}{\partial^{2}y}=-\frac{2}{h}\mu \varepsilon \left(x\right)$$

where v is the deformation of the flexible wall plate along the transverse direction, *y* is the coordinate length of the flexible wall plate along the transverse direction, Equation (11) indicates that the deflection on the central axis of the flexible wall plate is 0, Equation (12) indicates that the rotation angle along the transverse direction on the central axis is 0, and Equation (13) indicates that the bending moment at any position along the transverse direction is a function of the principal strain on the section in the primary bending direction.

According to the boundary conditions and solving the differential equation, it can be obtained that the beam function of flexible wall plate deformation along the transverse direction is

(14) $$v\left(y\right)=\frac{1}{h}k_{1}k_{2}\mu \varepsilon \left(x\right)y^{2}$$

where *k*_1_ and *k*_2_ are, respectively, the position influence coefficient considering the position influence of the section in the flexible wall plate, and the length-transverse ratio influence coefficient considering the ratio of the length and transverse of the flexible wall plate, and the calculation formulas are

(15) $$k_{1}=1-{\left(2*\frac{x-\frac{l}{2}}{l}\right)}^{2}$$

(16) $$k_{2}=1-6{\left(\frac{b}{l}\right)}^{2}$$

Based on the above functions, the OBF model of flexible wall plate is obtained as follows:

(17) $$w\left(x,y\right)=u\left(x\right)+v\left(y\right)$$

### 2.3. Deviation Analysis of the OBF Model

**Figure 3:**
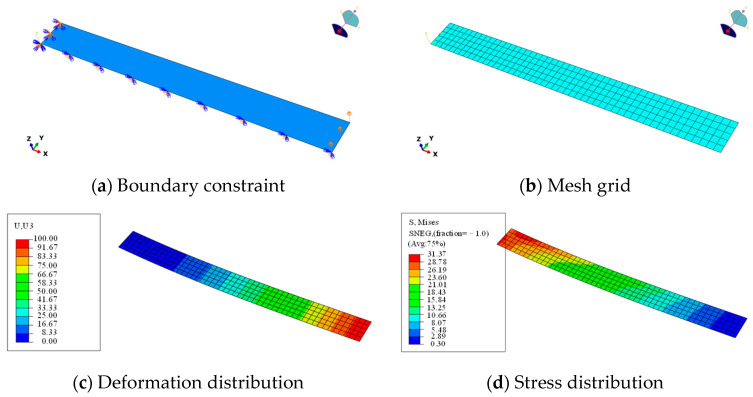
FE analysis of bending deformation of flexible wall plate.

The deformation of a flexible wall plate is analyzed by the finite element (FE) method and the OBF model. As illustrated in Figure 3, the FE model of the flexible wall plate was performed using ABAQUS 2016 software with the following structural parameters: 1000 in dimensionless length, 200 in dimensionless transverse, 20 in dimensionless thickness, 200 GPa in elastic modulus, and 0.3 in Poisson’s ratio. The plate was modeled with one end fixed and the other end subjected to a prescribed displacement of 100 (in consistent dimensionless units). The FE model employs a 4-node shell element (S4R) with a mesh size of 1/10 of the transverse dimension (i.e., 20 mm), resulting in 500 elements and 561 nodes.

**Figure 4:**
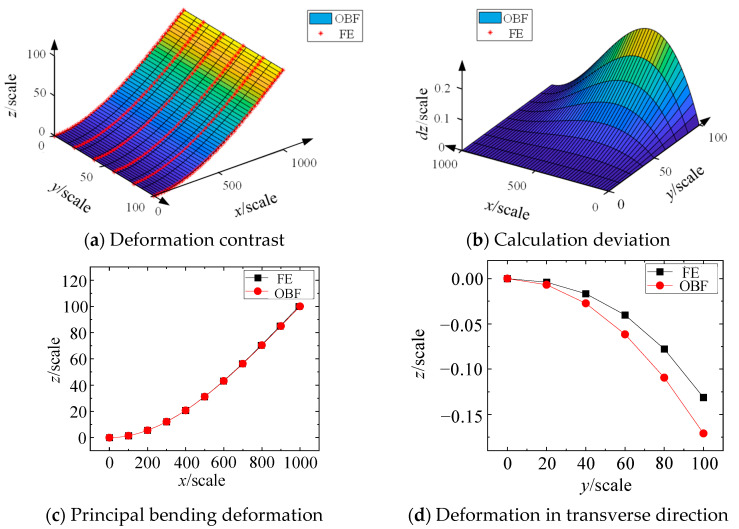
Comparison between OBF and FE results.

Figure 4 presents a comparison between the computational results obtained from the half-model FE and the OBF model, demonstrating excellent agreement between the two methods. The progressive color gradient from blue to red indicates increasingly minor to major deformation magnitudes, respectively. Figure 4c shows the displacement profiles along the central axis; the maximum discrepancy between the OBF-calculated deformation in the primary bending direction and the FE simulation results does not exceed 0.4. This error magnitude is significantly smaller than the maximum deflection (100) observed in the primary bending direction. The errors primarily arise from the assumptions of the theoretical model and the configuration details of the FE model. The comparison of transverse deformation profiles, presented in Figure 4d, reveals that the OBF model predicts slightly larger transverse deformations than the FE results. This difference stems from certain simplifications inherent in the OBF model. However, both computational approaches consistently demonstrate that the transverse deformations are substantially smaller than those in the primary bending direction, with the maximum transverse deformation being merely 0.15% of the maximum primary bending deflection. These findings collectively validate that the OBF model effectively characterizes both the primary bending and transverse deformations of flexible wall plates, thereby enabling accurate 3-D bending deformation analysis.

**Figure 5:**
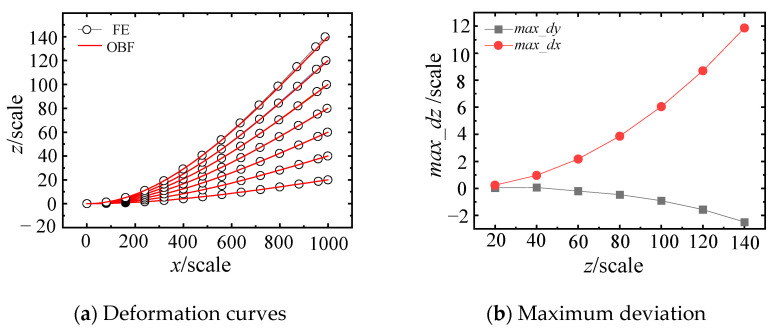
Comparative analysis of deformation results in the primary bending direction.

A systematic investigation was conducted to evaluate the effect of deflection on computational accuracy by employing both the FE and OBF models. The analysis encompassed seven discrete prescribed displacement cases (20, 40, 60, 80, 100, 120, and 140), with corresponding deformation profiles computed using both FE and OBF models, as presented in Figure 5. Figure 5a demonstrates that while the OBF solutions maintain satisfactory agreement with FEM benchmarks, the maximum deviation (*max_dz*) exhibits a monotonic increase with larger end deflections. Quantitative results in Figure 5b reveal that at the maximum deflection of 140, the deviation measures approximately 12 in the primary bending direction (*x*-axis) and 2.5 in the transverse direction (*y*-axis). The observed discrepancies primarily stem from the inherent limitations of the small-deflection theory underpinning the current OBF formulation, which neglects axial deformation components (particularly the *l* variation along the *x*-axis during bending deformation). Furthermore, the experimental data reveal a distinct nonlinear correlation between deflection magnitude and computational errors in both principal directions, characterized by accelerated error growth at higher deflection levels. As theoretically predicted by Equation (14), the progressive accumulation of errors in the primary bending direction (*x*-axis) propagates to the transverse deformation calculations, resulting in compounded deviations in the width-direction (*y*-axis) results.

**Figure 6:**
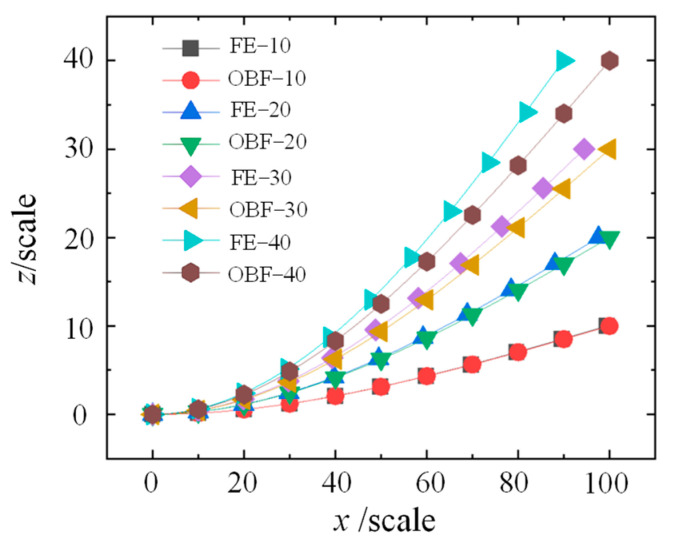
Comparison between OBF and FE results with dimensions of 100 in length.

Computational analyses were also performed on flexible wall plates with dimensions of 100 in length, 5 in width, and 5 in thickness. The free-end deflections were set to 10, 20, 30, and 40, respectively. The OBF calculation results were systematically compared with FE results, with the FE simulation parameters maintained consistent with the aforementioned conditions. As shown in Figure 6, the deformation results along the primary bending direction are presented, demonstrating good agreement between the OBF and FE results, which further validates the accuracy of the OBF model. However, as illustrated in Figure 6, the deviation between the OBF and FE results gradually increases with larger deflections, consistent with the observations in Figure 5b. This suggests that the OBF model, formulated based on small-deflection assumptions, requires further refinement to meet the computational accuracy requirements for large-deflection deformation analysis.

Considering the important influence of flexible wall plate on aerodynamic performance, under ideal conditions, the bending direction deformation of the flexible wall plate should be highly consistent with the designed aerodynamic profile. Therefore, it is necessary to modify the calculation of the large deflection deformation of the flexible wall plate in the primary bending direction based on the OBF model.

## 3. Solution of Large Deflection Deformation of Flexible Wall Plate

### 3.1. Elliptic Integral Method for Solving Large Deflection Deformation

**Figure 7:**
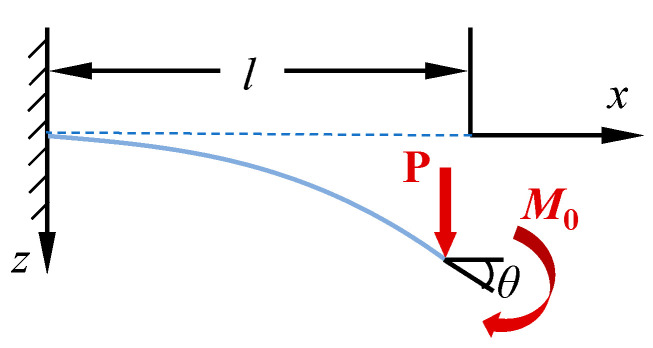
Large deflection deformation model of an elastic beam.

To improve the accuracy of calculating large deflection deformation with the OBF model, the elliptic integral (EI) method was used to solve the large deflection deformation problem of the flexible wall plate in the primary bending direction. As shown in Figure 7, the elastic beam with large deflection was selected as the beam function in the primary bending direction, the primary bending direction was the *x*-direction, and the forced displacement direction was the *z*-direction.

According to the Euler–Bernoulli beam theory, the relationship between the curvature and bending moment of a beam in plane bending is

(18) $$\frac{d\theta }{ds}=\frac{1}{\rho }=-\frac{M}{EI}$$

where the rotation angle *θ* is the angle between the tangent of the beam and the horizontal axis, the length *s* is the arc length coordinate along the neutral axis of the beam, *P* is the curvature radius, *M* is the bending moment at any point *x* in the primary bending direction of the beam, which is expressed as

(19) $$M\left(x\right)=M_{0}+P\left(l-x\right)$$

where *M*_0_ is the bending moment at the forced displacement and angle constraint end of the flexible wall plate, *P* is the concentrated force exerted by the forced displacement and angle constraint section of the flexible wall plate, and *l* is the length of the beam. The relationship between curvature and bending moment is

(20) $$\frac{d\theta }{ds}=-\frac{M_{0}+P\left(l-x\right)}{EI}$$

By differentiating both sides of Equation (20) with respect to *s*, as follows:

(21) $$\frac{d}{ds}\left(\frac{d\theta }{ds}\right)=\frac{P}{EI}\frac{dx}{ds}$$

For a micro-segment, the relationship among *dx*, *dz*, and *ds* is given as follows:

(22) dz=sinθds

(23) dx=cosθds

Let $\alpha =\sqrt{\frac{P}{EI}}$, Equation (21) can be rewritten as follows:

(24) $$\frac{d}{ds}\left(\frac{d\theta }{ds}\right)=\alpha^{2}\cos\theta$$

multiplying both sides of Equation (24) by dθ yields

(25) $$\frac{d\theta }{ds}d\left(\frac{d\theta }{ds}\right)=\alpha^{2}\cos\theta d\theta$$

the integration of Equation (25) gives the following:

(26) $$\int \frac{d\theta }{ds}d\left(\frac{d\theta }{ds}\right)=\alpha^{2}\int \cos\theta d\theta +C$$

(27) $$\frac{1}{2}{\left(\frac{d\theta }{ds}\right)}^{2}=\alpha^{2}\sin\theta +C$$

where *C* denotes a constant term. The boundary conditions of the forced displacement end are

(28) $$M\left(l\right)=-M_{0},\;\theta_{x=l}=\theta_{0},\;{\left(\frac{d\theta }{ds}\right)}_{x=l}=\frac{M_{0}}{EI}$$

where $\theta_{0}$ represents the rotation angle at the free end of the beam. Substituting the boundary conditions into Equation (27) yields the constant terms as follows:

(29) $$C=\frac{1}{2}{\left(\frac{M_{0}}{EI}\right)}^{2}-\alpha^{2}sin\theta_{0}$$

The relationship between ds and dθ can be expressed as follows:

(30) $$\sqrt{2}ds=\frac{1}{\alpha \sqrt{\sin\theta -\sin\theta_{0}+k}}d\theta$$

where $k=\frac{M_{0}^{2}}{2PEI}$. Furthermore, by integrating both sides of Equation (30) with respect to s over [0, *l*] and with respect to θ over $\left[0,\theta_{0}\right]$, we obtain the following:

(31) $$\sqrt{2}l=\int_{0}^{\theta_{0}}\frac{1}{\alpha \sqrt{\sin\theta -\sin\theta_{0}+k}}d\theta$$

Equation (31) represents the relationship between the arc length and angle for a rectangular thin plate. Considering the maximum out-of-plane deflection *z_l_* in the *z*-direction, and assuming each micro-segment satisfies Equations (22) and (23), we substitute Equations (22) and (23) into Equation (30) and integrate both sides to obtain the governing equation relating *z_l_* to $\theta_{0}$

(32) $$\sqrt{2}z_{l}=\int_{0}^{\theta_{0}}\frac{sin\theta }{\alpha \sqrt{\sin\theta -\sin\theta_{0}+k}}d\theta$$

Equations (31) and (32) may be reformulated as elliptic integrals in the following form [28]:

(33) $$\left\{\begin{array}{l} l=\frac{\sqrt{2}}{\alpha \lambda }f\\ z_{l}=\frac{\sqrt{2}}{\alpha \lambda }\left(f+\lambda^{2}e\right) \end{array}\right.$$

where λ, *f*, *e*, *t*, $\gamma_{1}$ and $\gamma_{2}$ are all elliptic integral solution parameters, $\lambda =\sqrt{\sin\theta_{0}-k-1}$, $f=F\left(\gamma_{1},t\right)-F\left(\gamma_{2},t\right)$, $e=E\left(\gamma_{1},t\right)-E\left(\gamma_{2},t\right)$, $\gamma_{1}=\sqrt{\frac{k}{-\sin\theta_{0}+k-1}}$, $\gamma_{2}=\sqrt{\frac{\sin\theta_{0}-k}{\sin\theta_{0}-k+1}}$, $t=\sqrt{\frac{\sin\theta_{0}-k+1}{\sin\theta_{0}-k+1}}$, $F\left(\gamma_{1},t\right)$ and $F\left(\gamma_{2},t\right)$ represent the first kind of complete elliptic integral, and $E\left(\gamma_{1},t\right)$ and $E\left(\gamma_{2},t\right)$ denote the second kind of complete elliptic integral.

*M*_0_ and *P* are obtained by solving Equation (33), and then the implicit bending equation of the elastic beam is obtained as follows:

(34) $$x=\frac{\sqrt{2}}{\alpha }\left(\sqrt{k-\sin\theta_{0}}+\sqrt{\sin\theta_{x}+k-\sin\theta_{0}}\right)$$

(35) $$z=\frac{\sqrt{2}}{\alpha }\frac{1}{\lambda }\left[f^{\prime }+\left(k-\sin\theta_{0}+1\right)e^{\prime }\right]$$

where $f^{\prime }=F\left(\gamma_{1}^{\prime },t\right)-F\left(\gamma_{2},t\right)$, $e^{\prime }=E\left(\gamma_{1}^{\prime },t\right)-E\left(\gamma_{2},t\right)$, $\gamma_{1}^{\prime }=\sqrt{\frac{\sin\theta_{x}+k-\sin\theta_{0}}{k-\sin\theta_{0}-1}}$.

### 3.2. Accuracy Analysis of EI Method

**Figure 8:**
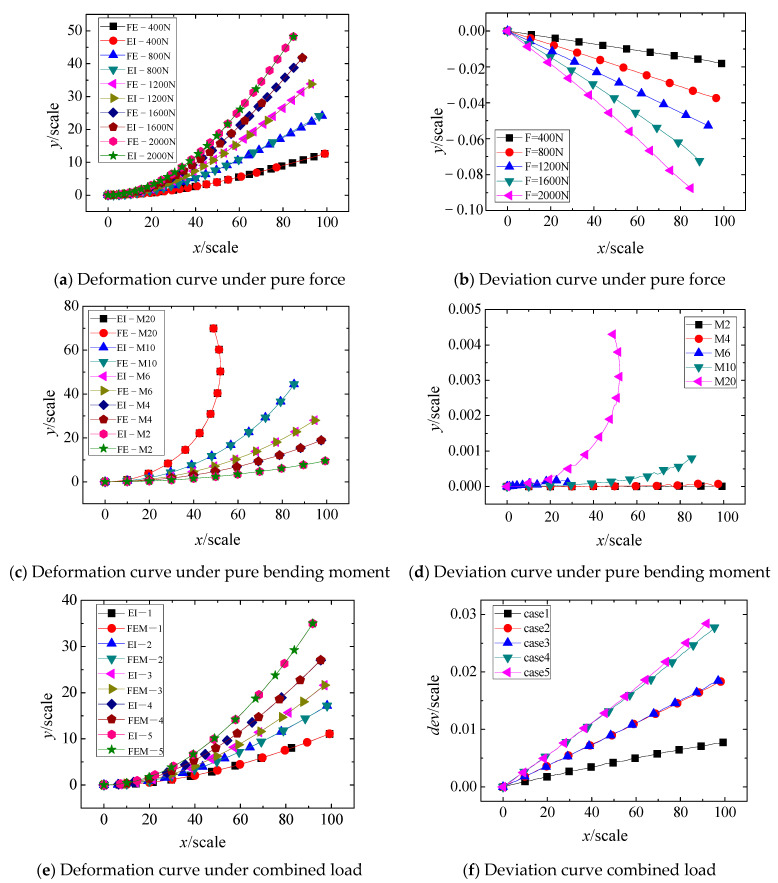
Comparison of calculation results between EI and FE methods in the principal bending direction.

To verify the accuracy of the EI method, the FE method was used to simulate the large deflection deformation of a cantilever beam. The cantilever beam is 100 in length, 5 in transverse and thickness, 200 GPa in elastic modulus, and 0.3 in Poisson’s ratio. The deviation between the EI method and the FE method under pure force load, pure moment load, and compound load was analyzed. Figure 8a,b, respectively, show the deformation profiles and deviation curves under pure force loading conditions (400 N, 800 N, 1200 N, 1600 N, and 2000 N applied at the free end). The results showed that the EI solution and FE results were highly coincident under different loads. With the increase of force load, the maximum deflections were close to half of the length of the cantilever beam. Although the deviation was gradually increasing, the deviation in *z*-direction was only 0.2% of the deflection, and the deviation in *x*-direction was 0.019, which was about 0.12% of the deformation in *x*-direction.

Figure 8c,d presents the deformation curves and deviation curves when the free end is only loaded with bending moment loads of 20 N·m, 40 N·m, 60 N·m, 100 N·m, and 200 N·m. The results show that the maximum deviation between the EI method and the FE method is approximately 0.0045, accounting for only 0.01% of the deflection value, indicating excellent agreement between the two methods.

For the case of combined force and bending moment loading on the cantilever beam, Table 1 summarizes five different loading conditions, with the corresponding deformation and deviation curves presented in Figure 8e,f. A comparison of the EI method and FE reveals that the discrepancy between the two methods increases with the applied load. Furthermore, the deformation error induced by the force component is significantly larger than that caused by the bending moment. Nevertheless, across all five loading cases, the maximum deviation remains within 0.11% of the peak deformation, demonstrating the robustness of the EI method.

The large deflection deformation of the flexible wall plate in the primary bending direction is obtained from Equations (34) and (35). Subsequently, the transverse deformation of the wall under large-deflection conditions is computed via Equation (14). The superposition of the primary bending deformation and the transverse deformation yields the OBF model for the large-deflection behavior of the flexible wall plate. Thus, the deformation in the primary bending direction and the transverse direction of the flexible wall plate with large deflection deformation is obtained.

## 4. Experimental Verification and Discussion

**Figure 9:**
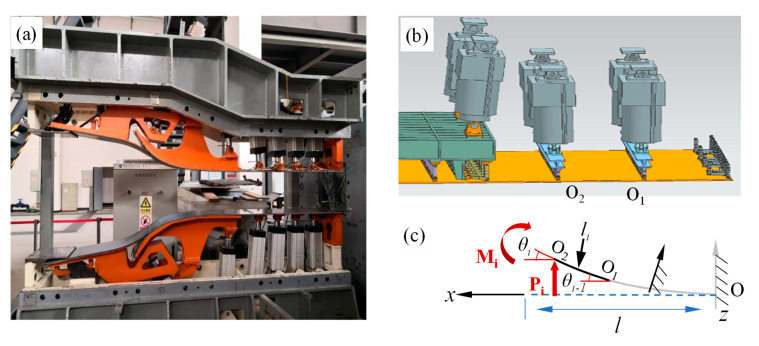
Schematic diagram of experimental setup and beam model. (**a**) Flexible nozzle. (**b**) Flexible wall plate. (**c**) Schematic of the plate segment deformation.

Large deflection deformation tests of the flexible wall plate are carried out to verify the accuracy of the calculation results of the OBF model. It should be noted that these validation tests focused on large-deflection deformations of the flexible wall plate induced by forced displacement of the driving rod, excluding aerodynamic loads or other practical engineering factors. Figure 9a illustrates the experimental platform for the flexible-wall nozzle. A spatial coordinate system was established with the nozzle exit as the origin, and deformations of the flexible wall plate were measured using a Leica AT960 (Leica, Wetzlar, Germany) absolute position laser tracker (measurement accuracy: 15 μm + 6 μm/m), which provides high precision. A prescribed displacement load was applied to the flexible wall plate, and experimental data were collected for rectangular plate segment *i* between the driving rod support points O_1_ and O_2_. As illustrated in Figure 9c, the segment was modeled as an elastically rotated beam with rotation angle *θ_i_*_−1_ at the fixed end and a known rotation angle *θ_i_* at the free end. The deformation of the plate segment was subsequently calculated using the OBF model to validate its accuracy in predicting large-deflection deformations.

**Figure 10:**
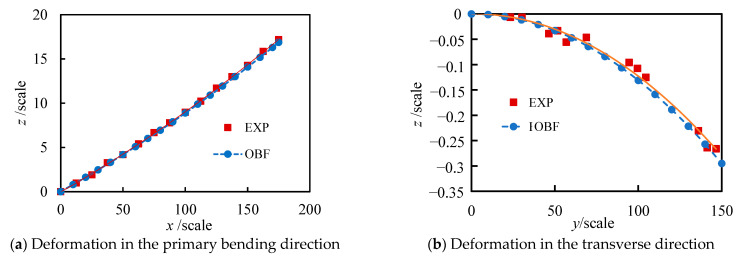
Comparison between the OBF model and experimental results.

The three-dimensional deformation of the *i*-th rectangular plate segment was analyzed using the OBF model and compared with experimental measurements, as presented in Figure 10. Figure 10a shows the deformation results along the primary bending direction (*x*-direction) obtained from both the OBF model and experimental measurements. Excellent agreement is observed between the computational and experimental results, with a maximum discrepancy of less than 0.7%. Figure 10b displays the deformation profile along the transverse direction at *x* = 75, revealing that the transverse deformation increases toward the edge regions. The maximum discrepancy between the OBF calculated and experimentally measured transverse deformations was less than 5.3%. The comparative results between OBF calculations and experimental measurements confirm that the OBF model can accurately predict the deformation of flexible wall plates under prescribed displacement loading.

## 5. Conclusions

This study investigated the deformation behavior of a flexible wall plate under complex boundary conditions. The OBF model was established to calculate the deformation of a flexible wall plate, and the deformation of small deflection and large deflection of the flexible wall plate in the transverse direction and primary bending direction was calculated. The following related conclusions can be drawn:

(1) Based on the small-deflection deformation of beams, an OBF model for flexible wall plates was established. The deformations of the flexible wall plate in both the principal bending direction and the transverse direction were calculated and compared with FE results. It was found that the OBF model agrees well with the FE results of the flexible wall plate under small deflections. However, the computational deviation gradually increases as the deflection grows.
(2) The large-deflection deformation in the principal bending direction was solved using the EI method, and the OBF model was accordingly modified. The results demonstrate that the modified OBF model significantly improves the accuracy of large-deflection deformation calculations. Under pure force loading, pure moment loading, and combined force-moment loading, the maximum computational deviation was found to be less than 0.2% of the deflection value.
(3) The modified OBF model was experimentally validated based on the deformation of a flexible wind tunnel nozzle. The results indicate that the modified OBF model exhibits good agreement with experimental data, with a maximum deviation of less than 0.7% in the principal bending direction (*x*-direction) and less than 5.3% in the transverse direction (*y*-direction).
(4) This study focuses on the deformation analysis of flexible wind tunnel wall panels. Future investigations will address two key aspects: (a) While the present work adopts prescribed support boundary conditions, subsequent research will explore boundary condition solutions for multi-support configurations in flexible wind tunnel walls; (b) The theoretical design approach presented here shows measurable deviations from actual structural profiles. To bridge this gap, subsequent research will combine online monitoring with the current methodology to reconstruct large-deflection deformations in multi-support flexible wall plates, enabling practical engineering applications.

## Figures and Tables

**Table 1:** Combined working conditions of force and bending moment.

Conditions	1	2	3	4	5
F (N)	200	400	400	600	600
M (N·m)	10	10	20	20	40

## Data Availability

The original contributions presented in this study are included in the article. Further inquiries can be directed to the corresponding author.
